# Trust, transparency, and disciplinary alignment in research funding evaluations: an illustrative case from the Norwegian AI Center call (2024–2025)

**DOI:** 10.3389/frma.2026.1874986

**Published:** 2026-06-26

**Authors:** Rune Johan Krumsvik, Marius Ole Johansen

**Affiliations:** Department of Education, Faculty of Psychology, University of Bergen, Bergen, Norway

**Keywords:** AI centers, committee bias, evaluation processes, framework, interdisciplinarity, procedural and domain-specific expertise, research funding, transparency

## Abstract

The allocation of national AI Centers in June 2025 represents a significant research-policy investment in artificial intelligence in Norway. The broad call, which explicitly included artificial intelligence in the field of education, resulted in 50 applications from large consortia across disciplines and sectors, with an overall rejection rate of 88%. This entailed a substantial aggregate investment of resources across the university, higher education, and research institute sectors, thereby presupposing evaluation processes characterized by a high level of disciplinary expertise, transparency, fairness, and procedural integrity. This article examines the evaluation process associated with the AI Center call, using one application (DLCAIC) as an illustrative case, analyzed through the lens of procedural knowledge, domain-specific expertise, and established research on research evaluation. It explores whether an investment equivalent to approximately half a full-time work year in proposal development can generate meaningful reflection, learning, and improved proposal competence—provided that the evaluation feedback is thorough and well-substantiated, even for unsuccessful applicants. This article is not presented as a conventional empirical study, but as a research-informed *Perspective* article based on an illustrative case. Its purpose is not to establish generalizable causal claims, but to use a concrete funding-process experience as a point of departure for discussing broader issues of trust, transparency, disciplinary alignment, and procedural fairness in research funding systems. The article further investigates whether committee bias and insufficient disciplinary and sectoral alignment between evaluation panels and application foci may lead to systematic distortions in assessment outcomes. The research literature demonstrates that such distortions may be associated with disciplinary preferences, institutional prestige, cognitive distance, and weakly calibrated evaluation criteria, particularly in interdisciplinary and sector-specific funding schemes. On this basis, the article propose a framework for assessing trust in research funding evaluations based on Panel-domain alignment, Transparency of expert selection, Calibration of self-declared competence, Consistency of written feedback, Actionability/feed-forward value, Procedural symmetry across applications, Disclosure of applicant positionality. The Perspective-article has clear limitations, as it is based on a single call, one application, and one evaluation process. With this caveat, it nevertheless addresses how differing “rules of the game,” weaknesses in panel appointment, competence profiles, and the application of evaluation criteria may undermine trust in the research funding system. The Perspective-article concludes by arguing for broader disciplinary representation in evaluation panels, clearer justifications for expert selection, and more transparent evaluation procedures as key measures to strengthen legitimacy and trust in the national research funding system.

## Introduction

What can reasonably be expected when applying for large-scale research funding schemes? Applicant communities assemble their strongest academic resources, invest substantial time and effort—often equivalent to more than half a full-time position—and submit proposals with the expectation of receiving a professionally competent, fair, and accountable evaluation conducted by experts with relevant domain-specific and sectoral expertise.

This principle is fundamental in academia and in society more broadly. A professor of education is not asked to assess a term paper addressing core biological questions, just as a biologist would not normally evaluate a paper in educational theory. Academic assessment presupposes alignment between the assessor's expertise and the object of assessment. The same principle applies in healthcare, where a patient with heart disease is examined by a cardiologist rather than an orthopedic surgeon, and in the legal system, where expert witnesses are appointed on the basis of specialized expertise in the core domain of the case.

In the evaluation of research proposals, general experience with assessment procedures alone is therefore insufficient. Evaluation panels must possess both **procedural knowledge**—an understanding of evaluation criteria, ranking, and decision-making processes—and **expert knowledge** within the disciplinary and sectoral core of the proposal. If either of these components is lacking, the basis for a precise and legitimate evaluation is weakened.

The question, then, is whether this principle is applied with sufficient consistency in the evaluation of research proposals. This article addresses that although evaluation committees face complex and demanding tasks, it is crucial that evaluation processes ensure professional competence, fairness, and procedural integrity for all applicants. This is not merely a matter of individual assessments, but of trust in the research system as a whole.

Norwegian academia is increasingly shaped by what *Nature* recently described as a defining condition of academic life: “Competition is a constant fixture of academic life. We compete for positions, promotions, publications and presentations. And we also compete for money, a necessary requirement if we are to continue taking part in the academic endeavor” (Schweiger, 2025, p. 1). Most academics operate within this competitive environment and generally accept proposal rejection, provided that the “rules of the game” governing research funding are clear, fair, and accountable. But are they always so in practice? This article addresses this question in light of the national AI Center call and the subsequent evaluation process in 2025.

The call and allocation of AI Centers in Norway in June 2025 represent a major research-policy milestone and are of considerable importance to the six strong consortia awarded national AI Center status^1^. The primary mandate of these centers is to advance national AI research over a 5–10-year period. For those consortia that were not funded, the process necessitates reflection, analysis, and a forward-looking orientation toward future funding opportunities. For the DLCAIC consortium, this has involved both critical self-assessment of the proposal's strengths and weaknesses and careful consideration of the evaluation feedback received from the assessment panel and the evaluation process as a whole. Consequently, the present article focuses primarily on the evaluation process rather than on the funding outcome itself.

From the outset, it was evident that the AI Center call^2^ was exceptionally broad in scope, enabling applications from a wide range of disciplines, professional fields, and AI orientations. Such broad calls and national AI initiatives require not only large consortia, high scientific quality, and strategic relevance among applicants, but also evaluation processes characterized by a high degree of expertise, legitimacy, and transparency. This is particularly important given the diversity of disciplinary foundations and AI orientations represented in the applications.

Our proposal—along with 43 others—was not awarded AI Center funding. With only six out of fifty applicant consortia funded, this outcome was largely determined by the financial framework of the call (NOK 1.3 billion) and the assessments conducted by the evaluation panels. The breadth of the call and the resulting rejection rate of 88% (44 of 50 applications) raise important questions regarding both the volume of academic labor invested and the evaluation of such large-scale competitive arenas. This is especially pertinent given the substantial collective effort invested by applicant consortia, with clear parallels to major funding schemes such as Centers of Excellence (SFF), Centers for Research-based Innovation (SFI), and Centers for Excellence in Education (SFU).

Those awarded AI Center status were assessed by the evaluation panels and the portfolio board of the Research Council of Norway (RCN) as the strongest consortia. Most applicants were well-aware of the high rejection rates associated with such calls and were therefore prepared for the likelihood of an unsuccessful outcome. DLCAIC likewise recognized rejection as a plausible result given the scale and competitiveness of the call. Nevertheless, this article does not address the rejection as such, but rather whether the rules of the competition were applied equally to all applicants and handled in a fair and accountable manner. At stake is the ability of the higher education sector, the research institute sector, and individual researchers to trust that the Norwegian research funding system operates in a reliable, fair, and accountable way.

Several developments over the past decade indicate that the research system in Norway, too, has significant room for improvement (Hesselberg et al., 2023; Stiftelsen Dam, 2025). International research on research evaluation shows that trust in research funding systems depends largely on equal rules for all applicants, perceived fairness, disciplinary alignment, and consistent application of evaluation criteria (Bornmann and Daniel, 2006; Langfeldt et al., 2020). When evaluation panels' expertise is insufficiently calibrated to the disciplinary and sectoral orientation of applications, the risk of various forms of committee bias increases, potentially undermining fairness principles, research quality, and system legitimacy (Langfeldt, 2006; Lamont, 2009). van Arensbergen et al. (2014) further demonstrate that social bias and group dynamics within evaluation panels may either weaken or strengthen the reliability and validity of assessments. It is, therefore, essential that portfolio boards and the RCN take all possible measures to ensure that applicants receive competent, fair, and accountable evaluations—particularly given the extensive academic labor invested in such funding processes both nationally (Stiftelsen Dam, 2025) and internationally (Schweiger, 2025).

The article is guided by three analytical questions:

1. To what extent is the evaluation feedback from the assessment panel consistent, stringent, and coherent?
2. To what extent can the evaluation feedback be used by DLCAIC to improve future research proposals?
3. To what extent is the composition of the evaluation panel adequately equipped to assess the proposal's orientation in accordance with the call and its evaluation criteria?

Addressing these guiding questions may, from a research-strategic perspective, yield important learning outcomes, enhance the quality of future research proposals, increase awareness of evaluation processes in large-scale funding schemes, and contribute to shedding light on underexplored aspects of the Norwegian research funding system.

## Background

Trust in the research funding system is closely linked to applicants' perceptions of whether different forms of evaluation bias have influenced assessment outcomes. What, then, does the research literature reveal about such biases? A substantial body of research demonstrates that committee bias in the evaluation of research proposals is a documented phenomenon with potentially serious consequences for both fair resource allocation and research quality. Several studies (e.g., Lee et al., 2013; Bol et al., 2018; Hesselberg et al., 2023; Stiftelsen Dam, 2025) show that evaluation committees do not always operate neutrally, but may be influenced by both explicit and implicit biases related to proposal orientation, applicants' gender, age, institutional affiliation, disciplinary background, and presentation style. Such biases are particularly salient in evaluation processes where expert judgement plays a central role and where evaluation criteria are weakly calibrated or inconsistently applied.

Bias may also arise in the form of **disciplinary bias**, whereby research topics, methodologies, or sector-specific knowledge that fall outside the committee's core expertise are systematically evaluated less favorably (Langfeldt, 2006). Research has further identified the so-called **Matthew effect**, whereby established researchers affiliated with prestigious institutions receive more favorable assessments than less established applicants with otherwise comparable proposals (Merton, 1968; Wennerås and Wold, 1997; Sandström and Hällsten, 2008; Huber et al., 2022). Such mechanisms may also manifest during the appointment of evaluation panels, particularly when expert selection is not blind and systematically favors established academic networks. To mitigate these forms of bias and nepotism, the literature recommends measures such as blinded review processes (Tomkins et al., 2017), increased methodological diversity within panels, awareness-raising regarding cognitive bias, and the use of more standardized and transparent evaluation criteria (Bornmann and Daniel, 2006).

Despite continuous improvements in quality assurance systems across national research funding regimes, the literature consistently shows that committee bias remains a complex and persistent challenge that can undermine fairness, accountability, and quality in research funding decisions. At the same time, there is relatively limited research on how evaluation panels themselves are appointed, even though this stage appears to be particularly critical for preventing bias later in the evaluation process. This lack of attention may be related to the fact that panel appointment is often handled administratively and not always perceived as an integral part of the formal evaluation process. It is therefore essential to examine whether this phase is subject to sufficiently robust quality assurance.

Evaluation panels must possess both **procedural knowledge**—that is, an understanding of evaluation criteria, ranking mechanisms, and decision-making procedures—and **expert knowledge** within the disciplinary and sectoral core of the proposal under review. If either of these elements is absent, the foundations for a precise and legitimate evaluation are weakened. Both the research literature and developments in national research systems suggest that greater attention should be directed toward this phase of the application and evaluation process in order to assess whether strengthened panel appointment practices could reduce committee bias. Studies show that when evaluation panels are composed of members with limited specialist expertise in the applicant's field, the risk of systematic undervaluation of proposals increases (Langfeldt, 2006; Lamont, 2009; Stiftelsen Dam, 2025). This is particularly evident in interdisciplinary projects or in cases where panels are required to assess work beyond their own disciplinary domains.

Such biases may be further amplified when committees are tasked with evaluating proposals within sectors where they lack contextual insight, in-depth understanding, and a relevant conceptual framework. As a consequence, original and innovative proposals may be perceived as unclear, unsystematic, or methodologically weak—even when they receive high quality assessments from domain-specific experts in other evaluative contexts^3^ (Porter and Rossini, 1985; Bammer, 2016). Meta-evaluations have likewise demonstrated that interdisciplinary projects tend to have lower average success rates, despite their considerable potential for innovative research (Rinia et al., 2001; Bromham et al., 2016). Whether elements of these dynamics were at play in the case of the DLCAIC consortium in connection with the national AI Center call is the question explored in the following sections.

### Methodological positioning

This article is positioned as a Perspective contribution rather than as a conventional empirical research study. The purpose is not to produce generalizable or replicable findings concerning research funding systems, but to use an illustrative case as a point of departure for a broader analytical discussion about trust, transparency, disciplinary alignment, and procedural legitimacy in research funding evaluations.

The analysis is informed by publicly available documents related to the Norwegian AI Center call, the written evaluation feedback received by the consortium, publicly available information regarding panel composition and competence profiles, and existing international research on peer review, research evaluation, committee bias, and interdisciplinary assessment. The article further draws on the authors' direct experience as applicants in the funding process.

The case should therefore be understood as an analytically illustrative and reflexive case rather than as a representative empirical sample. The article does not claim to establish causal relationships, empirically verify evaluator motivations, or document systematic bias across the funding system as a whole. Rather, the purpose is to identify structural conditions that, according to existing research literature, may increase the risk of perceived disciplinary bias, cognitive distance bias, and weakened trust in evaluation processes.

**Figure 1:**
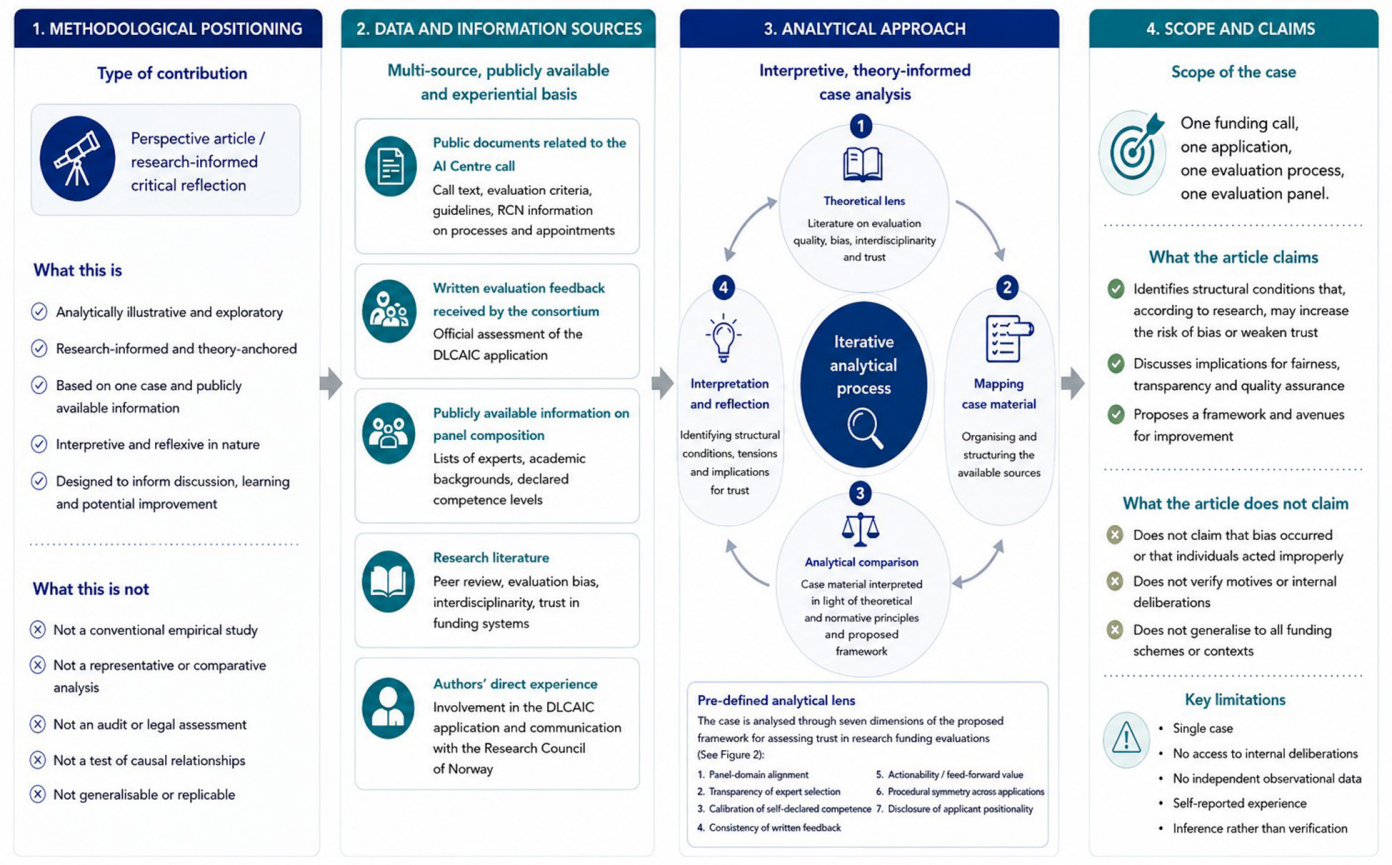
Methodological positioning of the present perspective article.

The perspective is consequently interpretive and exploratory in nature. The analysis is limited by the absence of access to internal panel deliberations, confidential assessment discussions, and independent observational data. The article should therefore be read as a research-informed contribution to ongoing discussions concerning transparency, legitimacy, and quality assurance in research funding systems, rather than as a formal evaluative audit of the specific funding process examined. Figure 1 shows an illustration of this process.

## Context

From a historical perspective, it is unprecedented that six Norwegian university-based research environments have been awarded large national AI Centers. This represents positive news for a wide range of sectors and signals a major national investment in artificial intelligence (AI). At the same time, the high rejection rate (88%; 44 of the 50 applications^4^ were rejected) reflects the highly competitive nature of centre funding. Although disappointing for the unsuccessful consortia, such outcomes may nevertheless stimulate emerging initiatives and smaller AI-center developments elsewhere in the country.

At the same time, important questions arise concerning the substantial number of academic work-years invested by the 44 applicant consortia that were not funded. On the one hand, this investment may be considered well spent if rejection is accompanied by evaluation feedback that contributes to improved proposal quality and more refined research-strategic thinking in future applications. On the other hand, the opposite effect may occur when evaluation feedback is vague, superficial, or insufficiently substantiated, thereby failing to enhance the quality of future proposals. If such patterns occur at scale, a persistently high rejection rate may become socio-economically unsustainable over time (Schweiger, 2025) and may ultimately contribute to stagnation in research quality. The research funding system is therefore highly dependent on evaluators' awareness of these dynamics and on evaluation panels and procedures being optimally designed to avoid such negative effects. Given that the Research Council of Norway (RCN) and its portfolio boards manage substantial public research funds on behalf of society, there is a particular responsibility to ensure that all rejected applications are evaluated in a professionally competent, fair, and accountable manner, accompanied by stringent, coherent, and well-developed feedback that applicant consortia can use constructively in future funding efforts.

Part of the learning process following the DLCAIC application^5^ (for which the first author served as consortium lead) in autumn 2025 involved analyzing such scenarios and critically examining the strengths and weaknesses of the proposal itself. The intention was to actively use the evaluation panel's feedback as a basis for learning. However, the feedback provided was so brief, inconsistent, and general that this proved difficult. General research on assessment shows that *feed up, feedback*, and *feed forward* are crucial for achieving learning outcomes from evaluations and for enhancing transparency and learning potential in assessment processes (Wisniewski et al., 2020). More specialized research on the evaluation of research proposals builds on the same principles (Hesselberg et al., 2023; Stiftelsen Dam, 2025). This raises questions as to whether the evaluation panel possessed sufficient awareness of this critical dimension of the evaluation process.

One possible explanation is that the brevity and generality of the feedback reflected limited understanding, insufficient knowledge, or inadequate engagement with the specific orientation of our proposal—consistent with what is described in the literature as **cognitive distance bias**. Alternatively, it may be that the panel faced such a high volume of applications that it lacked the capacity to provide more detailed feedback for each proposal. While the latter may offer a contextual explanation, it does not constitute a justification, as responsibility for ensuring adequate evaluation conditions ultimately lies with the commissioning body, in this case the RCN. In either scenario, the outcome was that our proposal did not receive sufficiently transparent or substantive feedback to support learning and improvement for future applications.

In order to identify potential learning points in our proposal and to better understand how different forms of evaluation bias may arise in large-scale funding schemes, we undertook a more in-depth review of the national and international research literature cited above, as well as comparable cases discussed in Norwegian academic media^6^ This tentative review provided insight into both applicants' own improvement potential in such processes and into possible systemic forms of committee bias, including disciplinary bias and cognitive distance bias. The Norwegian research funding system has long demonstrated awareness of such issues, and numerous measures have been introduced over recent decades to mitigate them. Nevertheless, structural conditions, traditions, historical trajectories, and portfolio preferences may still operate—consciously or unconsciously—as forms of tacit knowledge within the system.

Some of these dynamics became visible before and after the application deadline, when the RCN organized a series of seminars, webinars, and national conferences related to the AI Center call. Given the call's broad orientation, one might reasonably expect these events to reflect diverse foundational perspectives on AI across disciplines and to ensure broad representation in decision-making fora. However, a number of RCN-organized events in 2024 and 2025 did not reflect this inclusive breadth. Instead, they were largely characterized by AI conceptualizations rooted in STEM disciplines, thereby signaling implicit expectations regarding who the intended applicants were. This may partly reflect the historical dominance of STEM-based disciplines, major institutions, and AI research schools within the Norwegian AI landscape, which together form an epistemological backdrop for prevailing AI conceptualizations. Had the call been narrower and explicitly aligned with a STEM-oriented understanding of AI, this would have posed little concern. However, this was not the case. The call explicitly targeted a wide range of disciplines, professional fields, and sectors.

Such a broad call places a corresponding obligation on the RCN and its portfolio boards to ensure that accompanying events are inclusive and that competent and appropriate experts are appointed to evaluation panels calibrated to the orientation of each application. With a wide disciplinary spread, this task is inherently more demanding than in narrowly defined calls limited to, for example, STEM fields alone. This underscores the importance of both robust call design and institutional preparedness for managing diversity among applications and applicant environments.

In large-scale funding schemes, those with decision-making authority shape the overall orientation and priorities of national AI initiatives. An examination of the portfolio board^7^ responsible for the AI Center call reveals a clear dominance of STEM disciplines, with no representation from educational science or closely related fields. Given that portfolio boards establish key premises for funding schemes, this composition may—consciously or unconsciously—facilitate the emergence of **disciplinary bias** and **cognitive distance bias** at an overarching level.

A closer examination of the portfolio board and the RCN's appointment of evaluation panels shows that of the 43 experts^8^ appointed to assess applications, 36 were drawn from computer science, informatics, artificial intelligence, machine learning, information systems, natural language processing, quantum physics/technical physics, cybersecurity/cryptography, and software engineering. With 36 out of 43 experts (83.7%) representing STEM disciplines and 50 applications submitted, the probability that an evaluation panel would lack STEM representation is below 0.5%. This implies that STEM disciplines were structurally dominant within the evaluation system for the AI Center call.

The remaining experts comprised two from law/ethics/technology law, three from social sciences/media studies/anthropology, one from educational science, and one from statistics/finance/risk modeling. With only a single expert possessing an educational science background, representation of educational science in evaluation panels could either (a) occur in at most one panel if experts were not reused, or (b) depend entirely on whether the same expert was appointed to multiple panels. In both scenarios, educational science was structurally weakly represented. Consequently, non-STEM disciplines—such as educational science and pedagogy—had systematically low probabilities of representation in evaluation panels, increasing the risk of disciplinary bias. While it is possible that the predominance of STEM experts reflects an expectation that most applications would originate from STEM fields, procedural fairness requires that proposals from other disciplines, AI paradigms, professional fields, and sectors are afforded equivalent evaluation conditions through the appointment of relevant domain experts.

Applicants are entitled to expect that evaluation panels possess both procedural and expert knowledge, given that the call text functions as a legally binding framework governing the evaluation process. Failure by the RCN and portfolio boards to ensure this substantially increases the risk of disciplinary bias and deficiencies in both procedural and expert competence. When both the portfolio board and evaluation panels display a pronounced STEM orientation, this further suggests insufficient early-stage awareness of the potential side effects of such structural imbalances.

Given that our proposal was rooted in a fundamentally different disciplinary domain and AI conceptualization than STEM-oriented approaches, it cannot be ruled out that both cognitive distance bias and disciplinary bias were at play, whereby projects falling outside panel members' core expertise are systematically evaluated less favorably (Langfeldt, 2006). In particular, concerns arose regarding cognitive distance bias, as the evaluation panel may have lacked the disciplinary competence, conceptual framework, and methodological understanding necessary to assess the proposal's academic domain (Gulbrandsen and Langfeldt, 2004; Rafols et al., 2012; Hesselberg et al., 2023; Stiftelsen Dam, 2025). With only one of the 43 experts possessing educational science expertise, the risk of cognitive distance bias for applicants with such orientations was substantially increased. Responsibility for mitigating such risks rests with national research funding systems, and trust in these systems depends on their ability to prevent structural conditions that heighten the likelihood of such biases.

In the following section, we address specific aspects of the evaluation process through a case analysis based on the DLCAIC application, interpreted in light of the existing literature. The case is grounded in established theories of assessment (Wisniewski et al., 2020) and empirical research on peer review, research quality, and evaluation bias (Bornmann and Daniel, 2006; Lee et al., 2013; Langfeldt et al., 2020; Feliciani et al., 2022; Hesselberg et al., 2023; Stiftelsen Dam, 2025; Schweiger, 2025). Our approach is normatively delimited to the structural features of the evaluation process, including procedural and expert knowledge and implementation, rather than to the substantive quality of the proposal itself.

## Case

Because the RCN's evaluation panels were made public in June 2025, it was relevant to examine how the composition of our panel had been quality-assured and calibrated in relation to the disciplinary orientation of our proposal. Given the low transparency of the assessment we received—characterized by general, inconsistent, and vague feedback—we asked whether the evaluation panel was sufficiently equipped to assess our extensive and cross-sectoral application^9^. We questioned whether both **procedural knowledge** and **domain-specific expertise** were in place, and whether some form of **committee bias** may have weakened the integrity of the evaluation process. This concern was particularly salient because our interdisciplinary and cross-sectoral AI orientation within the field of education is distant from the STEM-oriented AI environments that have historically dominated Norwegian AI research. It is also important to note that our foundational AI perspective within educational research differs substantially from STEM-based AI paradigms, as it draws on the tradition of **Intelligent Tutoring Systems (ITS)** research, which emerged in the United States around 1970^10^ and has demonstrated strong effects in recent meta-analyses (Tlili et al., 2025).

Our review of the academic backgrounds of all members across all evaluation panels for the AI Center call revealed a strong predominance of STEM-related AI experts and, to the best of our knowledge, no evaluators with expertise in our ITS-oriented approach. Only one member across the evaluation panels had an educational science background—and, strikingly, this person was not included in our evaluation panel. This is a puzzling prioritization by the portfolio board and the RCN. The literature suggests that reducing such distortions requires greater disciplinary diversity within panels, the use of additional domain experts when necessary, blinded review, explicit attention to interdisciplinary evaluation bias, training in unconscious bias, and standardized and calibrated evaluation criteria. We do not know whether the RCN provided such training as part of the AI Center call; however, in general terms, these measures can contribute to fairer evaluation, greater awareness of self-declared competence levels, and increased trust in the research funding system. At the same time, studies indicate that not all training interventions are equally effective (Hesselberg et al., 2023), and that social bias and group dynamics, as well as imbalances between procedural and expert knowledge, may still influence panel processes. It is therefore important that such measures build on well-established evidence and recognized research on research evaluation.

As noted above, an especially prominent mechanism in the international literature is that **cognitive distance bias** and **disciplinary bias** may lead panels to favor research projects aligned with their own fields, disciplinary traditions, or methodological preferences, while rating projects outside their core expertise less favorably. Our review shows that our evaluation panel (with four members) consisted of one professor in information systems and digital transformation, one professor in digital innovation and marketing, one professor of computer science/informatics, and one adviser in data science. This indicates that the panel's core expertise lay in areas substantially different from educational science. This is concerning in light of the literature reviewed above. In addition, we note that one panel member appeared to hold a professional advisory role in data science without a PhD qualification. While such expertise may contribute valuable procedural or applied perspectives, this composition may raise questions regarding alignment with the RCN's stated competence requirements and with practices commonly observed in comparable funding schemes (Hesselberg et al., 2023; Stiftelsen Dam, 2025). Taken together, this panel composition substantially increases the risk of both cognitive distance bias and disciplinary bias.

To the best of our knowledge, members of the panel also lacked *sectoral knowledge* relevant to the educational field across health, defense, policing, and pedagogy—the sectors to which our proposal was explicitly oriented—thereby raising concerns about the quality assurance of panel appointment. It is plausible that the panel possessed strong procedural knowledge, but limited expert knowledge aligned with the proposal's substantive orientation.

Below, we outline more concretely why we consider that weaknesses relating to procedural and expert knowledge may have contributed to cognitive distance bias and disciplinary bias in the evaluation of our proposal.

1. **Alleged formal shortcomings in the appointment of the evaluation panel**

We believe that formal errors were made in the appointment of the panel tasked with evaluating our proposal. In particular:

**a. Failure to meet competence requirements**. The RCN's own competence requirements specify that panel reviewers should be active researchers with substantial scholarly output in both quantity and quality. Reviewers should ideally hold professorial competence, and the minimum requirement is senior/first-position competence^11^ We identified that one of the panel members appeared, at best, to hold only a master's degree, lacked a PhD and senior academic competence, was not an active researcher with a publication portfolio, and lacked both disciplinary and sectoral expertise. While this member may have had procedural knowledge, their domain-specific expertise was limited. This deviates from the RCN's stated requirements for panel members and, for example, led to the unusual situation in which this adviser was positioned to assess the scientific competence of a Nobel laureate in physics (given that one researcher in our consortium holds such a distinguished status).

**b. Absence of educational science core competence**. We consider it unlikely that a panel composed of experts in information systems and digital transformation, digital innovation and marketing, informatics, and data science is able to provide a professionally competent and valid assessment of a proposal anchored in an entirely different field—educational science and pedagogy. When all panel members lack educational science competence, the risk of multiple forms of committee bias increases.

**c. Lack of educational-science AI competence**. Our AI conceptualization in the proposal and in the educational research field is fundamentally different from STEM-oriented approaches, as it is grounded in ITS research initiated in the 1970s. Our review did not identify any panel member with AI competence specifically within ITS.

**d. Absence of sectoral competence**. The RCN requires that “the panel's combined competence shall cover the breadth of applications with regard to theme, discipline, and industry- and sector-specific knowledge along the axis from basic to applied research” (see text footnote 11). Based on what we have identified, the panel did not meet this requirement, as all four members lacked sectoral competence related to education across *health, policing, defense*, and *pedagogy*—the explicit orientation of the proposal.

**e. Capacity to evaluate scientific competence**. We also question how a panel consisting of experts in information systems and digital transformation, innovation and marketing, informatics, and data science can adequately assess the scientific competence of professors in *pedagogy, medicine, psychology, dentistry*, and *health sciences*, as well as a Nobel laureate in physics. The fact that the panel also rated our consortium below a threshold level on this dimension may likewise reflect committee bias. This underscores the future need for panel members with documented expertise and peer-review competence in the relevant disciplines, calibrated to the call's criteria and accompanied by full transparency in justifications for assessments of scientific competence.

**f. Limited interdisciplinary and cross-sectoral competence**. The RCN states that “panels are also composed with a view to handling interdisciplinary applications” (see text footnote 11). Our review did not identify evidence that panel members had experience with AI-based interdisciplinary and cross-sectoral proposal assessment of the kind our application represented.

**g. Disregard of proposed panel members**. In autumn 2024, all applicant environments were invited to suggest qualified panel members to support evaluation. To our knowledge, such suggestions were adopted in other cases. DLCAIC followed the RCN's recommendation and submitted 14 proposed qualified AI experts within the educational field from around the world (including from Stanford University), yet none were included in our panel. We also received no justification for why all 14 proposed experts were disregarded, despite the apparent absence of panel members with key sectoral competence, ITS-related AI competence, and educational expertise. This appears particularly puzzling given that the only expert across all panels with educational competence was not used in our evaluation panel. In light of the accumulation of unfavorable circumstances, this may raise broader questions regarding transparency, panel appointment procedures, and how evaluation systems seek to minimize the risk of unintended structural bias in expert selection processes (Wennerås and Wold, 1997; Sandström and Hällsten, 2008).

**h. Imprecise self-declared competence levels?** The RCN asks experts to indicate one of three competence levels for each application: **Specialist** [within primary area(s) of expertise], **Generalist** (general knowledge of at least one main subject), or **Minor** (only minor relevant expertise). Three of the four panel members declared themselves **Specialists** for our application, and one declared **Generalist**. In light of the above, this is striking. We, therefore, question whether panel members provided inaccurate or imprecise competence declarations, since it can be documented that one member did not meet formal education/research activity requirements and that none of the four possessed sectoral or educational expertise aligned with the proposal's cross-sectoral orientation. If so, the RCN cannot have sufficiently *validated* these competence declarations, which would appear inconsistent with its own quality assurance system.

2. **Limited transparency and consistency in the panel's assessment**

**a**. In fairness to the panel, it is possible that they did the best they could given their conditions and wrote their assessment with good intentions. Yet at this level, good intentions are not sufficient when applicants invest the equivalent of half a work-year in proposal development. The general and vague assessment provides little basis for understanding the proposal's specific strengths and weaknesses in a way that enables learning. The lack of internal consistency is particularly problematic, as illustrated by two excerpts from the assessment. The panel first wrote:

“Methodology: Its emphasis on large, scale, longitudinal and cross-sectional intervention studies highlights a robust methodological approach to understanding the impact of AI on educational outcomes. The center presents an ambitious research program, conducting literature reviews, applying RCT and MMR with clear anchoring in the current state of the art on learning technology related to LLMs across relevant domains. The methodology is clear and explained in sufficient detail, with the emphasis on the methodological aspects (...).”

Later, however, the assessment stated:

“There is too much emphasis on the scoping review, with a lack of quantitative elements. Incorporating metaanalyses on feedback studies or other quantitative approaches would have helped. It is unclear whether the work packages will utilize innovative research methods such as experimental designs, AI-supported qualitative research, or cross-sectional approaches.”

These statements are contradictory: the panel initially acknowledges robust quantitative and experimental components (including RCTs), yet later calls for precisely such approaches. This raises concerns about what occurred within the panel's deliberations. The assessment also appears to assume that scoping reviews lack quantitative elements (i.e., quantitative primary studies), which is incorrect. It further suggests that meta-analyses on feedback and AI-supported qualitative methods are missing, which is likewise inconsistent with the proposal. The panel's call for cross-sectional approaches is also noteworthy, given that several referenced and appended protocols for scoping reviews and preprints of AI-based case studies are cross-sectoral. These examples provide grounds to question the assessment's internal consistency and methodological precision.

Moreover, several statements are normative, weakly operationalised, and not clearly anchored in explicit evaluation criteria, making it difficult to determine what is missing, how different aspects were weighted, and why inconsistent formulations appear to outweigh the methodological robustness the panel itself initially described. The panel could have clarified these issues in the “Special points to consider” section and provided concrete guidance (*feed forward*), yet this field was left blank.

This lack of consistency, transparency, and actionable feedback may be interpreted in light of an imbalance between procedural and expert knowledge. The panel may have had strong procedural competence but limited domain-specific expertise aligned with the proposal's disciplinary and cross-sectoral orientation and AI conceptualization. It is also legitimate to ask whether the panel was familiar with foundational research on assessment feedback—much of which is situated within educational science (Wisniewski et al., 2020) and was referenced in our proposal.

3. **Potential shortcomings in the procedural-legal aspects of public calls**

**a**. We also experienced a lack of assessment of the criterion “Relevance to the call,” which is ambiguously formulated in the call text. The evaluation panel removed this criterion from our assessment without this being communicated clearly enough to applicants in the call documentation, apparently because of threshold procedures in the scoring rubric. As a result, substantial effort was invested in addressing this criterion in the proposal, even though it was not evaluated once an application fell below a certain score threshold.

**b**. Further concerns relate to discretionary judgement: STEM-oriented applicants were evaluated by the 36 (of 43) domain experts from their own sectors and disciplinary fields^12^, implying different evaluation conditions than those applied to us. This may constitute differential treatment and committee bias, for which the portfolio board and the RCN bear principal responsibility, raising procedural concerns.

**c**. Our concerns about the evaluation process were communicated to the RCN throughout summer and autumn 2025. We requested the appointment of a new evaluation panel (or the inclusion of additional experts) with educational science competence; this request was denied by the RCN. Legal experts have, on a general basis, suggested that our concerns may not have been handled adequately by the RCN^13^, which may further undermine trust in the research funding system.

Given that we experienced different “rules of the game” and identified multiple problematic features associated with cognitive distance bias and disciplinary bias, this naturally prompts reflection and learning for us as an applicant consortium. Equally important is that other applicants do not experience what we have experienced. It is, therefore, essential that the Norwegian research funding system and the RCN take all possible measures to prevent similar situations in the future—especially when the substantial labor invested in proposal development may, in practice, have been largely wasted. It is concerning that both our experiences and a number of other applicants and sources^14^ suggest that the RCN has faced a substantial portfolio of comparable cases^15^ over many years, highlighting the need to strengthen trust in the research funding system. This is particularly pressing given that several studies find that who evaluates an application may matter nearly as much as the quality of the application itself (Hesselberg et al., 2023; Stiftelsen Dam, 2025). Measures are needed to ensure high transparency in evaluation feedback, domain-specific disciplinary representation in panels, the use of additional external experts when required, and the development of evaluation procedures that explicitly account for cognitive distance in interdisciplinary and novel projects (Langfeldt and Kyvik, 2011; OECD, 2023). Such measures can strengthen trust rather than mistrust, and the RCN and portfolio boards play a crucial role in restoring and maintaining this trust.

### Toward a framework for assessing trust in research funding evaluations

Although trust is frequently described as a foundational element of research funding systems (Kunnskapsdepartementet, 2025), the concept itself is rarely operationalised in a sufficiently systematic manner within the literature on research evaluation. Existing studies have documented the importance of fairness, transparency, disciplinary alignment, and procedural integrity in peer review and funding decisions (Bornmann and Daniel, 2006; Langfeldt, 2006; Lee et al., 2013; Langfeldt et al., 2020). However, there remains limited conceptual work on how these dimensions may be integrated into a broader analytical framework for assessing trust in research funding evaluations.

Based on the literature reviewed in this article and the illustrative case presented above, we therefore propose a preliminary analytical framework for assessing trust in research funding evaluations. The framework is not intended as a formal measurement instrument, but rather as a conceptual and evaluative heuristic that may support future comparative research, policy development, and quality assurance processes in research funding systems.

**Figure 2:**
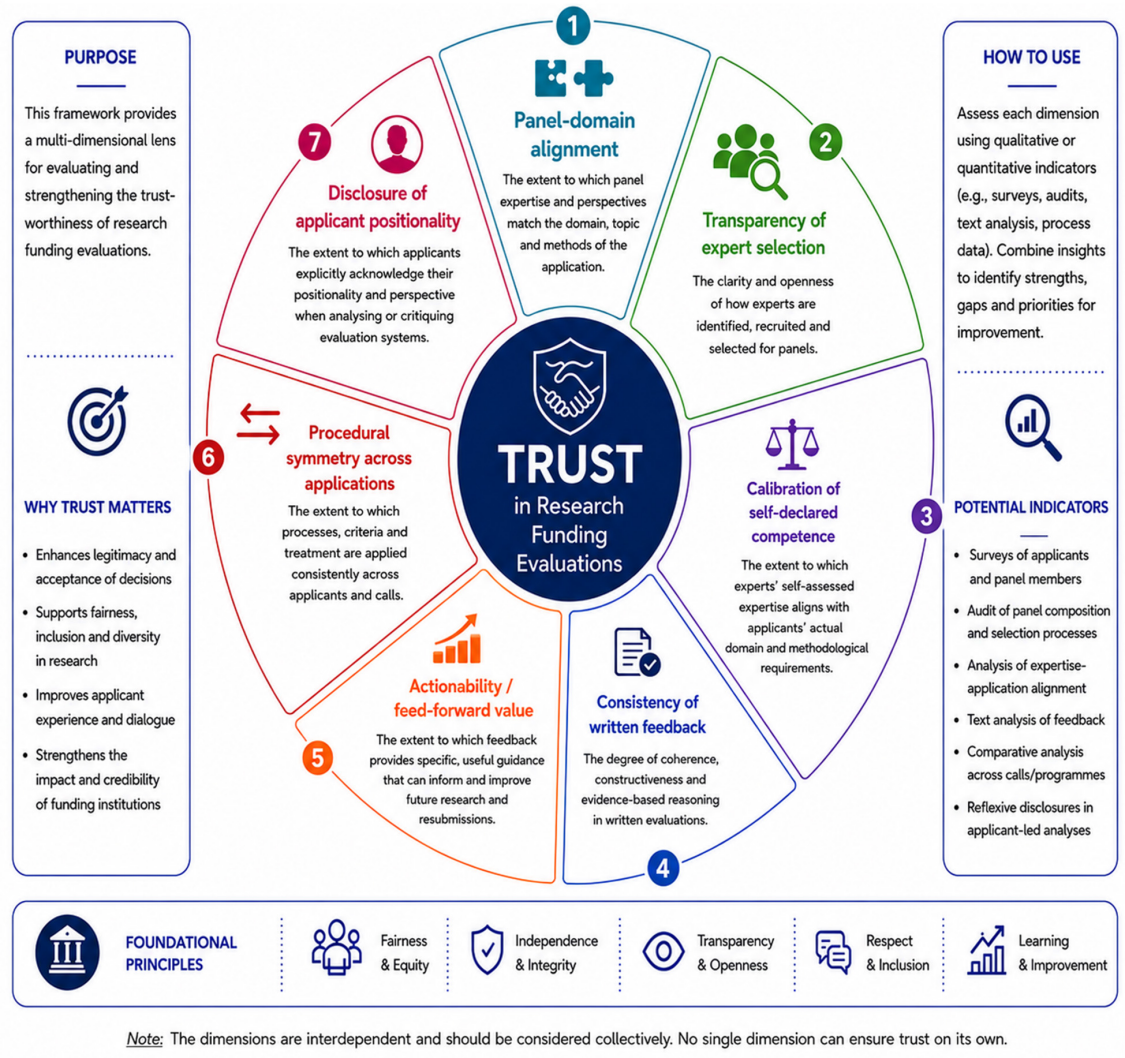
Toward a framework for assessing trust in research funding evalutions.

The framework consists of seven interrelated analytical dimensions (illustrated in Figure 2).

1. **Panel–domain alignment**

The first dimension concerns the degree of alignment between the disciplinary, methodological, and sectoral orientation of an application and the expertise represented within the evaluation panel. Research on interdisciplinary assessment demonstrates that weak alignment increases the risk of disciplinary bias and cognitive distance bias, particularly in broad funding calls involving heterogeneous research traditions (Langfeldt, 2006; Lamont, 2009; Bromham et al., 2016).

Trust in the evaluation system depends not only on procedural competence, but also on whether applicants perceive that evaluators possess sufficient substantive understanding of the proposal's disciplinary foundations, methodological traditions, and contextual orientation. Strong panel–domain alignment therefore constitutes a central prerequisite for legitimate and credible assessment.

2. Transparency of expert selection

The second dimension concerns the transparency and accountability of the processes through which evaluation panels are appointed. Research funding systems rely heavily on applicants' confidence that evaluators are selected according to professionally grounded and consistently applied criteria.

Transparency in expert selection includes clarity regarding competence requirements, the rationale for panel composition, handling of interdisciplinary applications, and the extent to which relevant sectoral expertise is represented. Increased transparency may reduce perceptions of arbitrariness, hidden preferences, or network-based selection mechanisms and thereby strengthen institutional legitimacy.

3. Calibration of self-declared competence

Many research funding systems require reviewers to self-assess their competence level in relation to individual applications. This process represents an important quality-assurance mechanism, yet relatively little research has examined how accurately such competence declarations are calibrated.

Trust may be weakened if evaluators declare specialist competence in areas substantially outside their disciplinary expertise, methodological familiarity, or sectoral knowledge base. Conversely, well-calibrated competence declarations may contribute to more reliable assessments, more appropriate allocation of applications to panels, and greater procedural legitimacy.

This dimension, therefore, concerns both the accuracy of self-declared expertise and the extent to which funding organizations validate and quality-assure these declarations.

4. Consistency and coherence of written feedback

The fourth dimension concerns the internal consistency, coherence, and analytical precision of written evaluation feedback. Research on assessment and feedback demonstrates that coherent and transparent feedback is crucial for legitimacy, learning, and applicants' ability to improve future proposals (Wisniewski et al., 2020).

Contradictory, vague, or weakly substantiated feedback may undermine confidence in the evaluation process and reduce the perceived legitimacy of funding decisions. This is particularly important in highly competitive calls where substantial academic labor is invested in proposal development. Trust is, therefore, influenced not only by funding outcomes themselves, but also by whether evaluation feedback appears analytically rigorous, internally coherent, and clearly linked to explicit evaluation criteria.

5. Actionability and feed-forward value

Evaluation feedback serves not only a summative function, but also a developmental one. The fifth dimension therefore concerns the extent to which evaluation feedback provides applicants with actionable guidance that may support learning and future proposal improvement.

Feed-forward value refers to whether applicants are able to identify concrete strengths, weaknesses, and areas for refinement based on the evaluation. When feedback is highly general, superficial, or insufficiently operationalised, its developmental value is substantially reduced.

In large-scale funding systems characterized by high rejection rates and substantial investments of publicly funded research time, the feed-forward quality of evaluations becomes particularly important from both a research-strategic and socio-economic perspective.

6. Procedural symmetry across applications

The sixth dimension concerns whether applicants are evaluated under equivalent procedural conditions across disciplinary and sectoral boundaries. Trust in research funding systems depends fundamentally on the perception that the “rules of the game” are applied consistently to all applicants.

Procedural symmetry includes issues such as equal access to domain-relevant expertise, consistent application of evaluation criteria, comparable treatment of interdisciplinary proposals, and equivalent opportunities for transparent justification of scores and rankings.

Where structurally different evaluation conditions emerge between disciplinary groups or proposal types, perceptions of procedural asymmetry may arise, potentially weakening trust in the fairness and legitimacy of the funding system.

7. Disclosure of applicant positionality

The final dimension concerns the explicit acknowledgment of applicant positionality and perspective when applicants themselves analyse or critique evaluation systems. In perspective-oriented analyses and reflexive studies of research funding processes, transparency regarding the authors' role and involvement is essential.

Disclosure of positionality does not invalidate critical analysis; rather, it allows readers to interpret arguments and interpretations within an explicitly acknowledged context. Such openness may strengthen analytical credibility and reduce ambiguity concerning potential interests, perspectives, or limitations.

### Integrative perspective

Taken together, these seven dimensions suggest that trust in research funding evaluations is not reducible to funding outcomes alone. Rather, trust emerges through the interaction between procedural integrity, disciplinary alignment, transparency, evaluative coherence, and applicants' perceptions of fairness.

The framework may therefore contribute to future comparative studies of research funding systems, meta-evaluations of peer review procedures, and the development of more transparent and robust quality-assurance mechanisms within national and international funding agencies.

## Discussion

The illustrative case presented in this article has clear limitations, yet it aligns with international research showing that interdisciplinary and sector-specific projects tend to have systematically lower success rates despite their high innovative potential (Bromham et al., 2016; Rinia et al., 2001). When evaluation panels—such as ours—seems to lack interdisciplinary, discipline-specific, and sector-specific competence, the likelihood increases that originality is misconstrued as lack of clarity or methodological weakness (Porter and Rossini, 1985; Bammer, 2016). This challenges fundamental principles of research quality, which are not universally defined but are context-dependent (Langfeldt et al., 2020). The absence of Norwegian experts with context-specific competence related to the educational field may also have reduced the panel's ability to understand the proposal's cross-sectoral orientation across four Norwegian sectoral contexts. OECD (2023), therefore, emphasizes the need for explicit mechanisms that address excessive cognitive distance and insufficient interdisciplinary competence in evaluation processes.

There is also a need for a broader epistemological and methodological discussion concerning foundational conceptions of AI across disciplines. Here, the AI Center call and the appointment of evaluation panels appear to assume a predominantly STEM-oriented understanding of AI, without adequately accounting for the fact that the educational field has engaged in AI development and AI research since the 1970s through the ITS tradition—an orientation that constituted the basis of our proposal (Koschmann, 2001; Krumsvik, 2023). When such mechanisms, uneven “rules of the game,” shortcomings, and structural evaluation errors occur, they not only affect individual assessments but also undermine trust in the research funding system as a whole.

## Summary and implications

In this *Perspective-*article, we have addressed the following guiding questions:

1. To what extent is the evaluation feedback from the assessment panel consistent, stringent, and coherent?
2. To what extent can the evaluation feedback be used by DLCAIC to improve future research proposals?
3. To what extent is the composition of the evaluation panel adequately equipped to assess the proposal's orientation in accordance with the call and its evaluation criteria?

In light of these guiding questions, the purpose of this article has been two-fold: first, to examine what applicant consortia may learn from such processes in order to strengthen future proposals; and second, to assess whether the call's broad orientation was reflected in the appointment of evaluation panels, and whether the evaluation process was quality-assured and fair in relation to the published evaluation criteria. By shedding light on these issues, we aim to identify learning points for applicant environments, strengthen future proposal quality, improve evaluation processes over time, and ultimately contribute to sustaining trust in the Norwegian research funding system.

The illustrative case indicates that the panel's feedback had clear shortcomings, particularly with regard to consistency, but also in terms of stringency and coherence. As a consequence, the feedback has limited utility for DLCAIC in improving future applications. The case further suggests that the composition of the evaluation panel substantially increased the risk of committee bias and was insufficiently equipped to assess the proposal's orientation and interdisciplinary foundation.

The article therefore suggests (see Figure 2) that the appointment process for evaluation panels requires stronger quality assurance in order to reduce the likelihood of committee bias—particularly cognitive distance bias and disciplinary bias—in large and broadly defined funding schemes where panel competence is insufficiently calibrated to the disciplinary and cross-sectoral orientation of applications.

A central problem associated with this form of committee bias is that the evaluation feedback intended to facilitate improvement and learning cannot fulfill that function when it is affected by multiple deficiencies. This is problematic in research terms, it undermines trust in the research funding system, and it also raises socio-economic concerns, given that the extensive work invested in proposal development represents publicly funded research time. More broadly, the issue concerns how research time in the higher education sector is used responsibly. When *Stiftelsen Dam* documents that 14.7 full-time equivalents were invested in unsuccessful applications in a far smaller call (Stiftelsen Dam, 2025), it is reasonable to assume that the corresponding figure was substantially higher for the AI Center call, given that 44 large consortia were rejected. It is therefore concerning if the RCN does not more robustly quality-assure the appointment of evaluation panels and the evaluation process itself when the resource investment is so extensive. In this context, procedural knowledge alone is insufficient; evaluation panels must also have robust expert knowledge aligned with each application's substantive orientation.

Relevant lessons can also be drawn from *Nature*'s recent critique of overly broad calls: “Avoid overly broad funding calls. When multiple disciplines compete for a limited pool of funds, the probability of success collapses. Agencies must create focused funding calls that target specific research areas” (Schweiger, 2025, p. 2). In light of this, the portfolio board and the RCN might have been better served by issuing multiple discipline- and sector-specific AI Center calls rather than a single, common call, given the consequences this broad approach appears to have had for the orientation of our proposal (and potentially for others).

The RCN and the portfolio board should, therefore, undertake a thorough review of the issues outlined above in order to reduce the likelihood of similar outcomes in future calls. From our perspective, a research funding system cannot sustain legitimacy if who evaluates an application proves to matter nearly as much as the quality of the application itself—as other studies also suggest (Hesselberg et al., 2023; Stiftelsen Dam, 2025).

## Limitations and positionality

The present article should be understood as a Perspective contribution based on an illustrative and analytically reflexive case, rather than as a conventional empirical study designed to produce generalizable or replicable findings. Its purpose is not to establish causal claims regarding the Research Council of Norway's evaluation system as a whole, but to use a concrete funding-process experience as a point of departure for discussing broader questions of trust, transparency, disciplinary alignment, procedural legitimacy, and evaluation quality in research funding systems.

The authors were directly involved in the DLCAIC application discussed in the article, and the first author served as consortium lead. This positional involvement is explicitly acknowledged and represents an important contextual and interpretive limitation. At the same time, the article does not involve commercial or financial conflicts of interest. The analysis is therefore written from an applicant perspective, with the interpretive limitations and reflexive considerations this entails.

Several additional methodological and analytical limitations should also be considered when interpreting the discussion. First, the analysis is based on a single funding call, one application, and one evaluation process. The case should therefore be regarded as analytically illustrative rather than representative of research funding systems more broadly. Consequently, the article does not claim that the dynamics discussed necessarily characterize other funding schemes, panels, or national contexts.

Second, parts of the analysis rely on publicly available information concerning panel composition, competence profiles, call documentation, written evaluation feedback, and related material. The authors did not have access to internal panel deliberations, confidential assessment discussions, reviewer interactions, or internal decision-making processes within the RCN. The article is therefore not in a position to establish intent, motivations, or actual bias among individual evaluators or decision-makers. Rather, the purpose is to illuminate structural conditions that, according to the research literature, may increase the risk of perceived disciplinary bias, cognitive distance bias, or weakened trust in evaluation processes.

Third, some analyses of disciplinary representation involve relatively small numbers, particularly in relation to educational science representation within the overall expert pool. As such, probability-related reflections should not be interpreted as empirical estimates of actual panel-selection outcomes, but rather as analytical illustrations of potential structural imbalances under particular assumptions regarding panel composition.

Fourth, although the authors invited the RCN to provide comments and clarifications regarding several of the issues discussed, the organization did not wish to contribute additional input beyond its formal responses. This may have limited the comprehensiveness of the account presented here and underscores the need for more systematic and comparative research involving multiple stakeholders, broader empirical material, and access to evaluation-process data.

Finally, the interpretive and exploratory nature of the article necessarily entails limitations related to selectivity, perspective, and analytical framing. Nevertheless, perspective-oriented analyses may still contribute value by identifying underexplored tensions, structural conditions, and conceptual challenges relevant to research funding systems, particularly in areas where systematic comparative research remains limited.

Taken together, these limitations suggest that the article should primarily be read as a research-informed contribution to ongoing discussions concerning evaluation quality, procedural legitimacy, interdisciplinarity, and trust in research funding systems, rather than as a formal evaluative audit of a specific funding decision or evaluation panel.

## Footnotes

1 Each of these AI centers was awarded approximately NOK 200 million.

2 The call text effectively functioned as the tender specification for this AI Center call, as the funding scheme was not announced through Doffin or similar procurement platforms, and was formulated as follows: https://www.forskningsradet.no/utlysninger/2025/forskningssentre-kunstig-intelligens/#tab.

3 See examples here: https://www.khrono.no/er-jeg-toppforsker-bunnforsker-eller-begge-deler/980616.

4 https://www.forskningsradet.no/contentassets/7f2a81ac18f54fa3bb6bd9a3ae198e49/ki-senter-soknader-jan2025.pdf—demonstrates.

5
https://www.uib.no/en/rg/dlc/44412/hva-er-digital-learning-communities-artificial-intelligence-centre-dlcaic

6 Example: https://www.khrono.no/ny-studie-tilfeldigheter-og-skjevheter-pavirker-hvem-som-far-forskningsstotte/994505.

7
https://www.forskningsradet.no/portefoljer/muliggjorende-teknologier/

8
https://www.forskningsradet.no/siteassets/tildelinger/2025/ki-sentre-2025-fageksperter—for-publisering-pa-nettsiden.pdf

9 The application was oriented toward the educational domain within the health, defense, police, and education sectors and involved 22 university environments and researchers, as well as 25 partner organizations. The consortium comprised 16 educational researchers, 7 medical researchers, 5 odontologists, 7 psychologists, 4 health researchers, and 6 interdisciplinary researchers representing the four sectors. The proposal itself consisted of five work packages, with a particular focus on artificial intelligence, education, and health-related themes.

10 Intelligent Tutoring Systems (ITS) were originally developed as early as around 1970, beginning with the doctoral dissertation of Jamie R. Carbonell, alongside contributions from his father, the well-known machine learning researcher Jamie G. Carbonell (who later developed the language model XLNet, which in 2019 significantly outperformed the widely recognized language model BERT). Our collaborator in the DLCAIC center application, Dr Etienne Wenger, was part of this research environment during the 1980s and published a book on Intelligent Tutoring Systems in 1987, at a time when AI technology was still relatively immature and limited in capability. As a result, Wenger and many of the most influential AI/ITS researchers of that period—such as Lauren Resnick, Allan Collins, James Greeno, Herbert Simon, John Seely Brown, Seymour Papert, Richard Mayer, and Kurt VanLehn—experienced several so-called “AI winters.” Nevertheless, their research in AI and educational technology constitutes a foundational component of the historical knowledge base of AI in education. Moreover, a number of meta-analyses in recent years have demonstrated that ITS performs very strongly (Tlili et al., 2025). It was this AI orientation that underpinned our AI Center application, rather than a STEM-oriented conception of artificial intelligence.

11
https://www.forskningsradet.no/finansiering/forskningsorganisasjoner/kompetanse-samarbeidsprosjekt/behandlingsprosess/#rekruttering

12 The 43 experts who evaluated the applications: https://www.forskningsradet.no/siteassets/tildelinger/2025/ki-sentre-2025-fageksperter—for-publisering-pa-nettsiden.pdf.

13 The Research Council of Norway (NFR) has been given several opportunities to respond to our remarks and points of appeal, but has stated that our objections are not upheld, referring to them as matters falling within its discretionary judgement. We also invited NFR to engage in a dialogue regarding our concerns, which they likewise declined.

14 For example: https://www.khrono.no/er-forskningsradet-tilliten-verdig/1011695.

15 Here are some of the cases: 1, 2, 3, 4, 5, 6.
